# Biochemical response of *Rhodotorula mucilaginosa* and *Cladosporium herbarum* isolated from aquatic environment on iron(III) ions

**DOI:** 10.1038/s41598-019-56088-5

**Published:** 2019-12-20

**Authors:** A. Cudowski, A. Pietryczuk

**Affiliations:** University of Białystok, Faculty of Biology, Department of Water Ecology, 15-245 Białystok, Ciołkowskiego 1J, Poland

**Keywords:** Iron, Fungal physiology

## Abstract

The objective of the paper was to determine the influence of iron(III) ions on the growth and metabolism of fungi commonly occurring in waters: the yeast *Rhodotorula mucilaginosa* and filamentous fungus *Cladosporium herbarum*. Cells of *R*. *mucilaginosa* were shown to absorb the most iron(III) ions at a concentration of 1 mg/L iron(III) ions. Yeast cells showed a considerable increase in the content of proteins and monosaccharides, as well as biomass growth. At higher concentrations of iron(III) ions, the yeast limited the intake of iron(III) ions, and a decrease in the basic metabolites in cells was observed, as well as an increase in the secretion of such metabolites into the medium. Moreover, the activity of antioxidant enzymes increased in the fungal cells, suggesting that iron(III) ions have a toxic effect. Simultaneously, even at high concentrations of iron(III) ions in the medium, no decrease in the yeast biomass was recorded. It seems therefore that the potentially pathogenic *R*. *mucilaginosa* will likely be present in waters moderately contaminated with iron(III) ions. It can be useful as a water quality bioindicator. A considerably higher capacity for the biosorption of iron(III) ions was recorded for the filamentous fungus *C*. *herbarum*. Defensive mechanisms were observed for *C*. *herbarum*, which were manifested in a substantial increase in the content of proteins and monosaccharides, as well as an increase in the activity of antioxidant enzymes, particularly under the influence of high concentrations of iron(III) ions. Moreover, it was evidenced that in the filamentous fungus, iron(III) ions limited the extracellular secretion of metabolites. These results suggest that the fungus can actively accumulate iron(III) ions and therefore eliminate them from the aquatic environment. It can be useful in water treatment processes, which has a significant impact on water ecology.

## Introduction

The occurrence of microfungi has been so far recorded in all types of aquatic ecosystems in the world. In addition to autochthonous species, for which water is a natural living environment, many species of fungi are supplied to waters with surface runoff or are of anthropogenic origin. A large number of fungi supplied to water depths cause diseases in animals and humans^1^. Species of pathogenic or potentially pathogenic fungi that are most frequently isolated from aquatic ecosystems belong to the following genera: *Candida* sp., *Cryptococcus* sp., *Rhodotorula* sp., *Aspergillus* sp., *Trichophyton* sp., and *Cladosporium* sp.^2^ An important role of fungi involves their capacity for the biotransformation of xenobiotics^3^ and heavy metals^4^ supplied to the aquatic environment. They can contribute to reducing the effects of anthropogenic stress and improving water quality. According to the available literature, certain heavy metals (nickel, lead, copper, zinc) may limit the development of aquatic fungi^5–7^, and other heavy metals (e.g., cadmium) can reduce their toxic effect with the participation of fungi belonging to hyphomycetes^8^. However, contemporary literature does not provide information on the influence of iron(III) ions on the metabolism of filamentous fungi and yeasts. Many scientists have proposed including fungi in the group of bioindicators of anthropogenic transformations of the aquatic environment used in monitoring the ecological state of aquatic ecosystems^9–11^ and the sanitary state of waters^12,13^; thus, these studies are important.

Iron ions can undergo transformation depending on the prevailing physicochemical conditions and the presence of microorganisms^14^. In an alkaline environment, with good oxygenation, iron ions precipitate, and under acidic conditions with hypoxia, its compounds dissolve. Such iron behavior is influenced by a number of factors such as: water reaction, oxidation-reduction potential, concentration of organic matter, type of minerals (especially clay) and the presence of some cations (e.g. iron, aluminum) or anions e.g. silicates^15^. The presence of iron(III) ions is very important from the point of view of the functioning of aquatic ecosystems. Already at a low concentration (140 μg/L), iron(III) ions activate slow reproduction of cyanobacteria and an increase in the concentration of microcystins by 30% on average^16^. This is one of the factors leading to the development of cyanobacterial blooms and the intensification of eutrophication processes, which have become a global problem observed in lakes, seas, and rivers around the globe^17^.

Due to the above, the objective of the paper was to determine the effect of iron(III) ions on the growth and metabolism of potentially pathogenic fungi commonly occurring in waters: the yeast *R*. *mucilaginosa* and filamentous fungus *C*. *herbarum*. *Rhodotorula* sp. especially *R*. *mucilaginosa* are known as opportunistic pathogens that are particularly dangerous for immunocompromised patients^18,19^. *C*. *herbarum* can cause black spot on cereals^20^ and is one of four *Cladosporium* sp. that cause disease in humans^21^. Insight into such correlations will permit finding the answer to the following question: do iron(III) ions activate or limit the development of these pathogens in surface waters, or perhaps are the studied fungal species able to remove iron(III) ions from the environment? Answering these questions will constitute an important step towards including the species in the group of bioindicators for determining the sanitary state of waters.

## Results

The content of iron(III) ions in cells of *R*. *mucilaginosa* increased 9-fold in comparison to the control under the influence of 1 mg/L iron(III) ions and 11-fold under the influence of 100 mg/L iron(III) ions. A much higher increase in the content of iron(III) ions in cells was recorded in the case of *C*. *herbarum*, which was able to accumulate 82–330 times more iron(III) ions in comparison with the control as a result of treatment with 25–100 mg/L iron(III) ions, respectively. Moreover, in the presence of high concentrations of iron(III) ions in the medium (25–100 mg/L), their absorption by the yeast was inconsiderable (biosorption at a level of 2% and 1% respectively), whereas the filamentous fungus absorbed approximately 51% of the iron(III) ions present in the medium (Table 1).

**Table 1:** Biosorption of iron(III) ions by filamentous fungi cells (*Cladosporium herbarum*) and yeast cells (*Rhodotorula mucilaginosa*) (mean value ± SD).

The concentration of Fe^3+^ introduced into the medium [mg/L]	*Rhodotorula mucilaginosa*			*Cladosporium herbarum*		
	the concentration of Fe^3+^ in the medium [mg/L]	the concentration of Fe^3+^ in the cell [mg/g]*	*biosorption of Fe*^*3+*^ *from the medium* [%]	the concentration of Fe^3+^ in the medium [mg/L]	the concentration of Fe^3+^ in the cell [mg/g]*	*biosorption of Fe*^*3+*^ *from the medium* [%]
0	0.00	0.09 (±0.001)	—	0.00	0.12 (±0.003)	—
0.25	0.14 (±0.001)	0.21 (±0.008)	*44*.*0*	0.09 (±0.003)	0.19 (±0.004)	*64*.*0*
1	0.19 (±0.003)	0.85 (±0.01)	*81*.*0*	0.83 (±0.02)	0.70 (±0.03)	*17*.*0*
5	4.89 (±0.01)	0.21 (±0.009)	*2*.*8*	4.06 (±0.05)	0.70 (±0.002	*18*.*8*
25	24.5 (±0.90)	0.39 (±0.007)	*2*.*0*	12.3 (±0.06)	9.87 (±0.04)	*50*.*8*
100	99.0 (±2.30)	1.06 (±0.02)	*1*.*0*	49.1 (±1.10)	39.6 (±1.11)	*50*.*9*

*Concentration in mg per 1 g fresh weight of the fungus.

**Figure 1:**
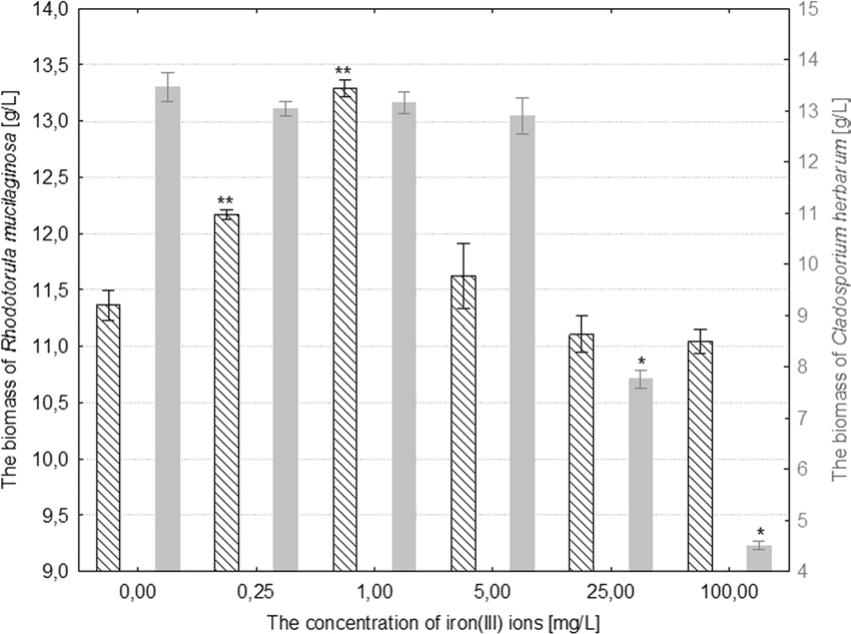
The effect of different concentrations of iron(III) ions on the biomass of *Rhodotorula mucilaginosa* and *Cladosporium herbarum* (n = 12 ± SD). (*) Indicates statistically significant differences between treatment with iron(III) ions and the control (p ≤ 0.001). (**) Indicates statistically significant differences between treatment with iron(III) ions and the control (p ≤ 0.005).

**Figure 2:**
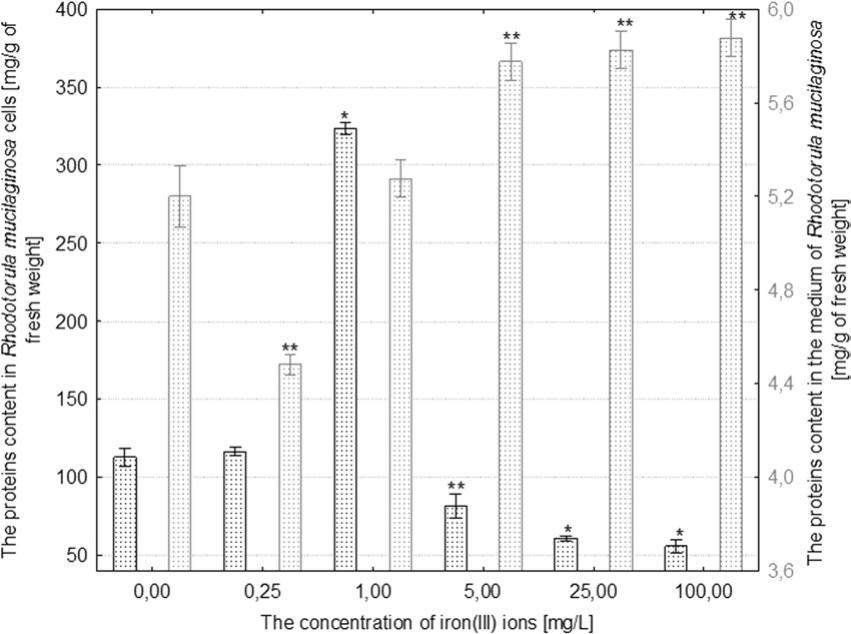
The effect of different concentrations of iron(III) ions on proteins content in the cells and in the culture medium of *Rhodotorula mucilaginosa* (n = 12 ± SD). (*) Indicates statistically significant differences between treatment with iron(III) ions and the control (p ≤ 0.001). (**) Indicates statistically significant differences between treatment with iron(III) ions and the control (p ≤ 0.005).

An inconsiderable increase in the biomass of *R*. *mucilaginosa* was only recorded under the influence of 0.25 mg/L and 1 mg/L iron(III) ions, which increased the biomass by 8% and 15%, respectively, in comparison to the control culture (p ≤ 0.005). Treatment of the aforementioned fungal species with iron(III) ions at concentrations over 5 mg/L caused no statistically significant changes in its biomass in comparison to the control (Fig. 1). In the case of *C*. *herbarum*, after adding 5 mg/L iron(III) ions to the medium, no statistically significant changes were recorded in the biomass of the fungus in comparison to the control sample. Higher concentrations of ions of this metal (25–100 mg/L), however, induced a substantial decrease in the biomass by 44–66%, respectively, in comparison to the control (p ≤ 0.001) (Fig. 2).

**Figure 3:**
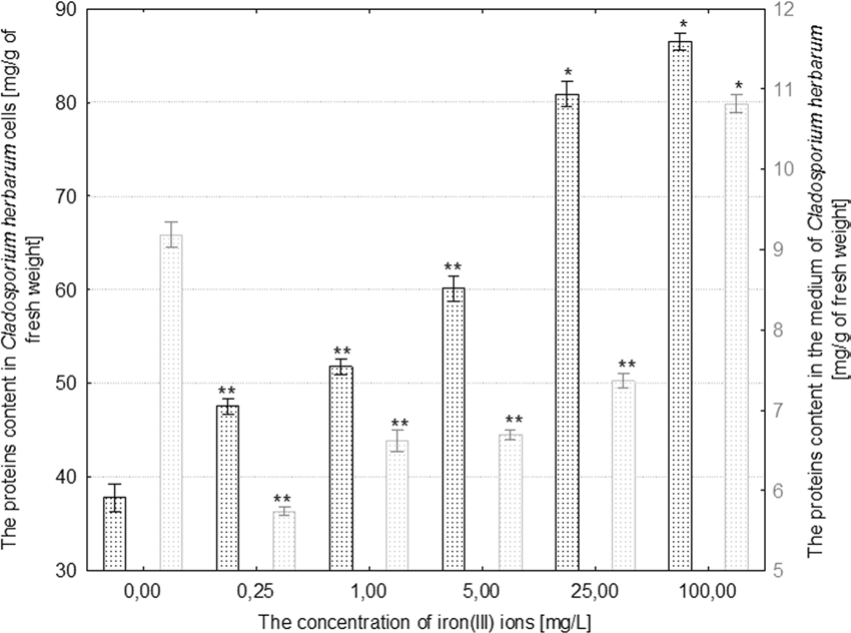
The effect of different concentrations of iron(III) ions on proteins content in the cells and in the culture medium of *Cladosporium herbarum* (n = 12 ± SD). (*) Indicates statistically significant differences between treatment with iron(III) ions and the control (p ≤ 0.001). (**) Indicates statistically significant differences between treatment with iron(III) ions and the control (p ≤ 0.005).

The highest increase in concentration (by 175% in comparison to the control) of proteins in cells was recorded in *R*. *mucilaginosa* treated with 1 mg/L iron(III) ions. The higher concentrations of iron(III) ions induced a substantial decrease in the content of proteins in the fungal cells in comparison to control, max. by 50% in the case of treatment with 100 mg/L iron(III) ions (Fig. 2). Moreover, yeast cells growing under the influence of over 5 mg/L iron(III) ions secreted an average of 20% more proteins into the medium in comparison with the control culture (Fig. 2). In the case of *C*. *herbarum*, the lowest increase in concentration of cellular proteins (by 20% in comparison to control) was recorded under the influence of 0.25 mg/L iron(III) ions, and the highest (by 132% in comparison to control) was recorded in the presence of 100 mg/L iron(III) ions (Fig. 3). Iron(III) ions added to the medium at concentrations from 0.25 to 25 mg/L slowed the process of protein secretion in *C*. *herbarum*. Only the highest applied concentration of these metal ions (100 mg/L) caused a small increase in the concentration of proteins in the medium by 13% in comparison to the control culture (p ≤ 0.001) (Fig. 3).

**Figure 4:**
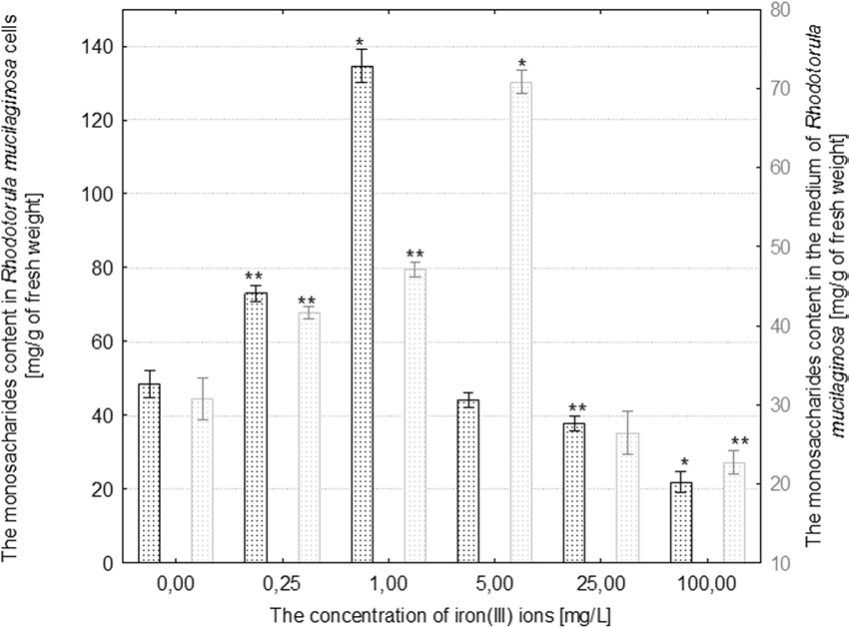
The effect of different concentrations of iron(III) ions on monosaccharides content in the cells and in the culture medium of *Rhodotorula mucilaginosa* (n = 12 ± SD). (*) indicates statistically significant differences between treatment with iron(III) ions and the control (p ≤ 0.001). (**) indicates statistically significant differences between treatment with iron(III) ions and the control (p ≤ 0.005).

**Figure 5:**
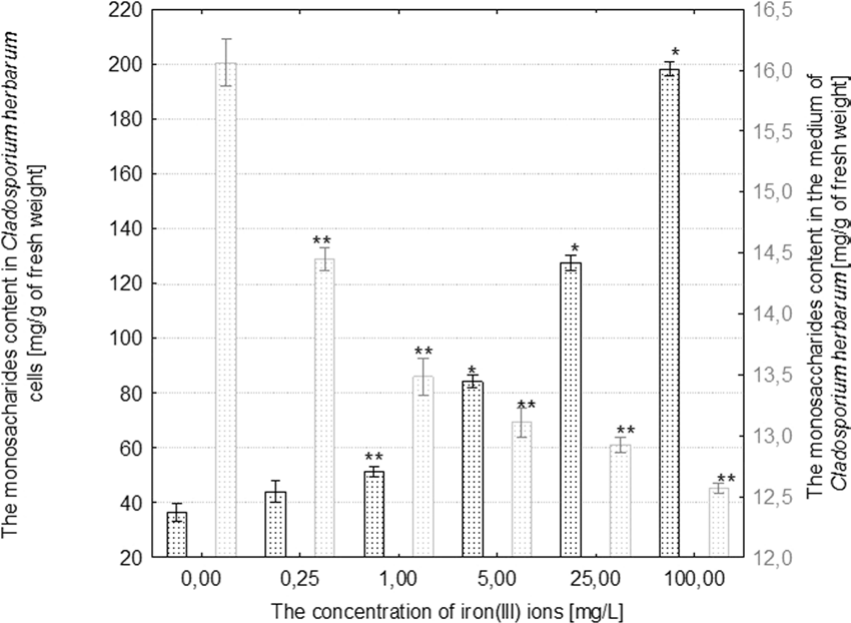
The effect of different concentrations of iron(III) ions on monosaccharides content in the cells and in the culture medium of *Cladosporium herbarum* (n = 12 ± SD). (*) Indicates statistically significant differences between treatment with iron(III) ions and the control (p ≤ 0.001). (**) indicates statistically significant differences between treatment with iron(III) ions and the control (p ≤ 0.005).

The treatment of *R*. *mucilaginosa* with 0.25–1 mg/mL iron(III) ions induced a statistically significant increase in monosaccharides in fungal cells in comparison to the control. Under the influence over 25 mg/mL iron(III) ions, a significant decrease in the monosaccharide content in fungal cells was observed in comparison to the control (p ≤ 0.001) (Fig. 4). The concentration of monosaccharides in the medium increased as a result of treating *R*. *mucilaginosa* with 1–5 mg/L iron(III) ions, while the highest concentrations of these metal ions induced a significant decrease in the secretion of these metabolites (Fig. 4). The entire range of iron(III) ion concentrations caused an increase in the content of monosaccharides in cells of *C*. *herbarum*. The highest concentration of reducing sugars in fungal cells in comparison to the control, which was more than a 5-fold increase, was recorded in the culture treated with 100 mg/L iron(III) ions (Fig. 5). Moreover, it was evidenced that iron(III) ions applied at concentrations of 0.25–100 mg/L considerably inhibited the secretion of reducing sugars by cells of the filamentous fungus into the culture medium (Fig. 5).

**Figure 6:**
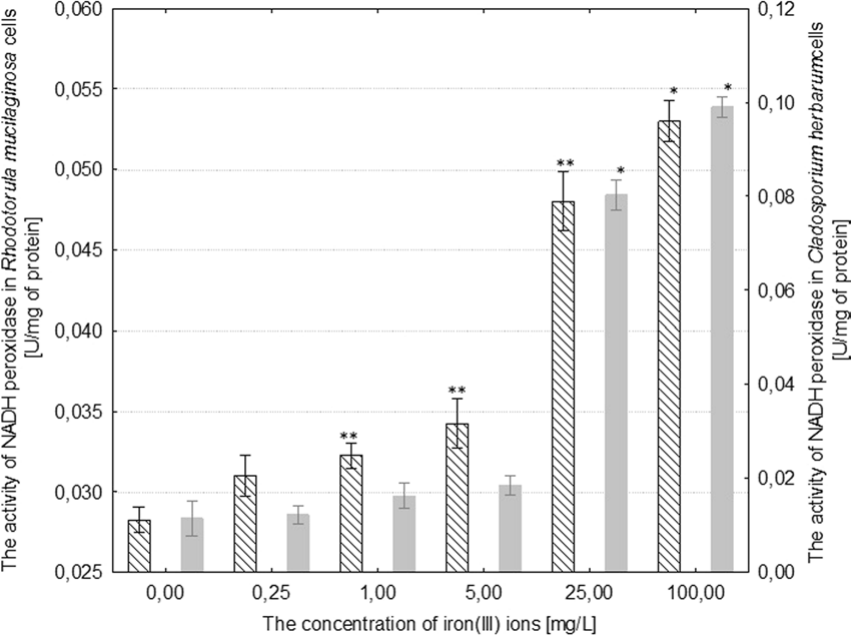
The effect of different concentrations of iron(III) ions on NADH-dependent reductase activity in the cells of *Rhodotorula mucilaginosa* and *Cladosporium herbarum* (n = 12 ± SD). (*) Indicates statistically significant differences between treatment with iron(III) ions and the control (p ≤ 0.001). (**) Indicates statistically significant differences between treatment with iron(III) ions and the control (p ≤ 0.005).

**Figure 7:**
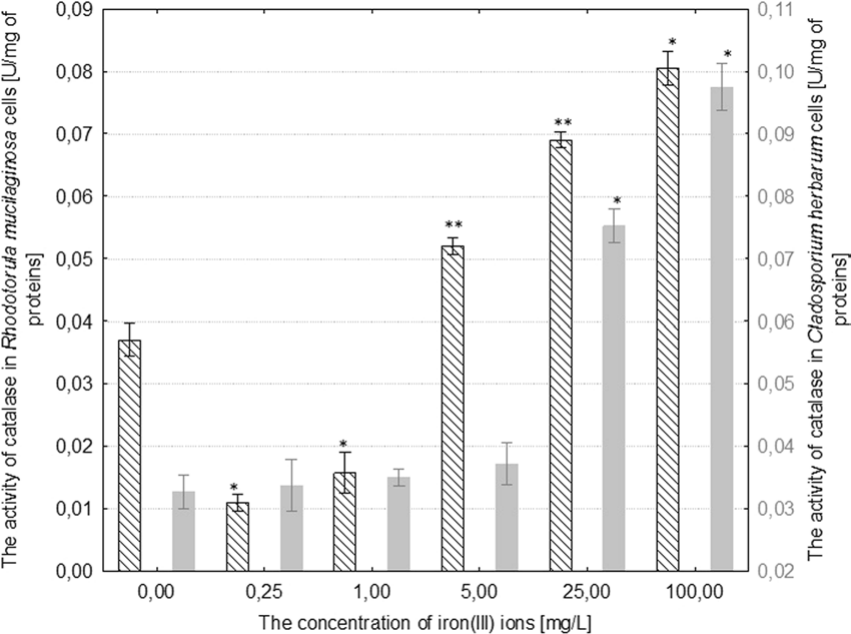
The effect of different concentrations of iron(III) ions on catalase activity in the cells of *Rhodotorula mucilaginosa* and *Cladosporium herbarum* (n = 12 ± SD). (*) Indicates statistically significant differences between treatment with iron(III) ions and the control (p ≤ 0.001). (**) Indicates statistically significant differences between treatment with iron(III) ions and the control (p ≤ 0.005).

**Figure 8:**
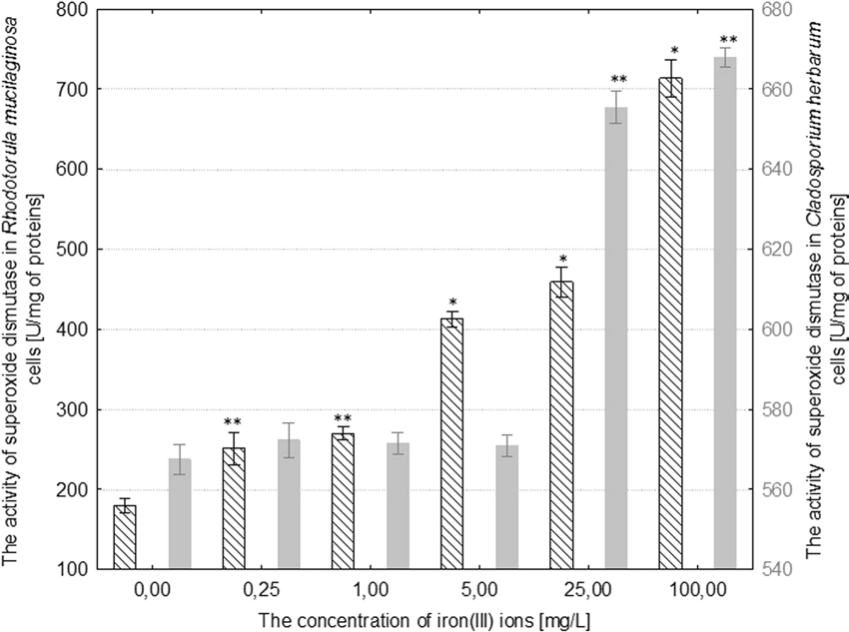
The effect of different concentrations of iron(III) ions on superoxide dismutase activity in the cells of *Rhodotorula mucilaginosa* and *Cladosporium herbarum* (n = 12 ± SD). (*) Indicates statistically significant differences between treatment with iron(III) ions and the control (p ≤ 0.001). (**) Indicates statistically significant differences between treatment with iron(III) ions and the control (p ≤ 0.005).

The activity of NADH-dependent reductase increased by 7–67% in comparison to the control as a result of treating *R*. *mucilaginosa* with iron(III) ions at concentrations of 1–100 mg/L. The activity of catalase in the fungal cells increased by 37–110% in comparison to the control culture under the influence of 5–100 mg/L iron(III) ions, respectively. Moreover, the activity of superoxide dismutase increased by 62–182% with regard to the control as a result of treating *R*. *mucilaginosa* with 0.25–100 mg/L iron(III) ions (Figs. 6–8). The addition of 5 mg/L iron(III) ions to the medium of *C*. *herbarum* did not cause a significant change in the activity of the three analyzed enzymes in comparison to the control. The concentration of iron(III) ions over 25 mg/L induced a significant increase in the activity of all of the analyzed antioxidant enzymes: NADH-dependent reductase by approximately 775%, catalase by 145% and superoxide dismutase by 18% in comparison to control (Figs. 6–8).

## Discussion

The intensive development of industry and agriculture over recent years^22^ has resulted in a continuous increase in the contamination of aquatic ecosystems with heavy metals, particularly Fe, Cu, Zn, or Pb. This elicits considerable changes in the species structure of aquatic microorganisms^6^. In addition, the contamination with heavy metals, in turn, results in the necessity of searching for microorganisms capable of accumulating or transforming heavy metals and hence the inactivation of heavy metals and elimination of their toxic effect on the environment.

On the one hand, iron is a microelement that limits the growth and development of aquatic microorganisms. On the other hand, after exceeding a certain threshold of its concentrations, the element can have a toxic effect^22^. The filamentous fungus *C*. *herbarum* was much more sensitive to the effect of high concentrations of iron(III) ions than the yeast *R*. *mucilaginosa*. Moreover, it seems that iron is not a microelement necessary for the growth and functioning of *C*. *herbarum* because no significant biomass growth of the fungus was observed under the influence of ions of the metal in contrast to yeast. However, the obtained results suggest that iron ions are necessary for the proper growth, development, and virulence of potentially pathogenic yeasts, as previously determined in *Candida albicans*, which even developed mechanisms of obtaining iron bound to proteins of the host^23^. Moreover, fungi from the genus *Rhodotorula* synthesize rhodototorulic acid, fulfilling the function of the iron transporter in tissues^24^. Our analyses also showed that high concentrations of iron(III) ions do not cause a statistically significant decrease in the biomass of the fungus below the control values. This suggests that a high abundance of pathogenic yeasts can be expected in waters contaminated with iron compounds. This is confirmed by data showing that strongly polluted waters are dominated by potentially pathogenic yeasts^13^, whereas many species of filamentous fungi do not develop in waters with high concentrations of biogenes, organic matter, and heavy metals^3,4,9,25–27^. Moreover, it was evidenced that the abundance and species diversity of zoospore fungi decrease in waters with high concentrations of iron(III) ions^28^. Species of filamentous fungi capable of development in waters contaminated with heavy metals were distinguished by good properties of bioaccumulation of metals (Zn, Cd, Pb, Ni) in their cells^5,29,30^. The bioaccumulation capacity of fungi is due to the presence of a cell wall that has different structures in filamentous fungi and yeast^31^. The diverse structure of fungal cell walls results from the presence of various functional groups, which show different sorption possibilities^32,33^.

Considerably higher biosorption of iron(III) ions by *C*. *herbarum* in comparison to yeast suggests that it is a fungus capable of accumulating iron(III) ions. This may be because the main component of the filamentous cell wall is chitin, which has an amide group^32^ in contrast to yeasts. Nitrogen, a component of the amide group, has a free electron pair that can form coordination bonds with metal cations, including iron(III) ions^34^. Microorganisms significantly affect the bioavailability of iron. The mechanism of the enzymatic oxidation of iron(II) by microorganisms occurs on their cell walls in an acidic environment. In turn, the essential element of the iron(III) bioreduction process is the presence of chelating-forming microorganisms that produce metabolites capable of forming iron chelate complexes^15^. Thanks to the activity of microorganisms, numerous redox processes occur, which in combination with fungal organic matabolits play a very important role in biogeochemical cycles of metal circulation, causing on the one hand an increase in their toxicity, and on the other their inactivation^35,36^.

*C*. *herbarum* can cause inactivation and thus reduce the availability of metal ions in the aquatic environment. This is confirmed by the fact that at high initial concentrations of iron(III) ions in the medium, a significant increase in the content of proteins and monosaccharides was recorded in cells of *C*. *herbarum*. It was evidenced that heavy metals such as Cd, Zn, Hg, Cu, or Ni induce the expression of the *smtA* gene in cells of the microorganisms, and *smtA* genes encode metallothioneins, i.e., proteins responsible for binding ions of heavy metals^22^. In cells of the filamentous fungi *Heliscus lugdunensis*, *Flagellospora curta*, and *Fontanospora fusiramosa* treated with ions of Cd, Ni, or Cu, above average synthesis of glutathione, phytochelatins, and proteins rich in thiol groups was observed, which were also responsible for binding ions of heavy metals^5,37,38^. Moreover, metallothioneins are also known to fulfil a protective function against the effect of free oxygen radicals, the synthesis of which is generated by heavy metals^39^. Therefore, an increase in the protein content in *C*. *herbarum* cells treated with high concentrations of iron ions can also be a mechanism activated in response to the appearance of free oxygen radicals.

A similar regularity has also been reported for monosaccharides in *C*. *herbarum* cells. Glucose and its derivatives are known to fulfil the function of signal particles in fugal cells in response to the activity of various environmental stress factors, causing, among others, the activation of the cascade of MAP kinases, which influence the expression of genes encoding stress proteins and proteins controlling cell divisions and differentiation^40,41^. Considering also an increase in the content of proteins, an increase in the concentration of monosaccharides in cells of *C*. *herbarum* treated with high concentrations of iron(III) ions presumably leads to the activation of the expression of genes responsible for the synthesis of stress proteins. It was also evidenced that certain heavy metals induce the mechanism of programmed cell death (PCD) in aquatic fungi, which is related to the synthesis of free oxygen radicals^42^. This is in accordance with the results of our own research showing that *C*. *herbarum* treated with high concentrations of iron(III) ions have significantly increased antioxidant enzyme activity compared to the control values and a simultaneous reduction in the fungal biomass in the medium. No similar mechanisms were recorded in yeast cells. Additional evidence suggesting that high concentrations of iron(III) ions are toxic for *R*. *mucilaginosa* is that the iron(III) ions caused a decrease in the content of monosaccharides in cells of these fungi in comparison to control. Iron(III) ions are necessary for the proper metabolism of yeast, and probably for the development of its virulence, which confirms that at lower concentrations of iron(III) ions, the content of both saccharides and proteins in the cell is maintained at the control level. It was evidenced that glucose and its amine derivatives induce virulence in another yeast, such as *Candida albicans*^40,41^. Therefore, it seems that waters with high concentrations of iron(III) ions should show particular sanitary threats.

Treatment of both filamentous and yeast-like fungi with iron(III) ions also has a considerable effect on the secretion of metabolites into the medium. The hindered secretion of proteins and monosaccharides in filamentous fungi is probably related to the mobilization of these metabolites in cells. The proteins are synthesized among others for the purpose of binding iron(III) ions intensively absorbed by fungal cells^38^. In turn, the monosaccharides act as messengers of the molecular signal to the cell nucleus in response to a stress factor^40^. Fungi exposed to stress caused by heavy metals are known to secrete low-molecular polypeptides. They are primarily nucleases, proteinases, and lysozyme^43^. Therefore, it seems that for yeast, 5 mg/L iron(III) ions is already toxic and causes defensive responses to stress. Proteins secreted into the medium probably bind ions of the metal in the environment or transform the metals into nontoxic forms, and as a result, reduce their mobility and avoid their penetration into cells. Extensive metabolic activity of fungi associated with the active production of various exometabolites, causes significant changes in the distribution of metal ions in the aquatic environment and in their mobility, bioavailability, and toxicity^44^. One of the mechanisms of the response of cells to stress caused by heavy metals is the synthesis of reactive oxygen species (ROS) and the related oxidative stress^7,45^. It was evidenced that in cells of the yeast *R*. *mucilaginosa*, the increase in activity of selected antioxidant enzymes was recorded in the presence of higher concentrations of iron(III) ions in the medium. This suggests a toxic effect of iron(III) ions on the fungal cells. Moreover, these concentrations of iron(III) ions in the medium in which yeast grew results in stress responses (a decrease in the content of proteins and monosaccharides and increase in the activity of antioxidant enzymes), and absorption of ions of the metal by cells considerably decreases. At low concentrations of iron(III) ions in the culture environment (up to 1 mg/L), yeast intensively absorbs iron(III) ions that cause no stress response because no intensive increase in the enzymatic activities of antioxidant systems or decrease in the content of basic metabolites was observed in *R*. *mucilaginosa* cells. ROS can also develop as a result of proper metabolic transformations, such as byproducts or intermediates of oxygen metabolism^39^, which is why a slight increase in the activity of enzymes above the control value can occur. Another mechanism was observed in the case of the filamentous fungus. Iron(III) ions at a concentration of up to 5 mg/L, similar to yeast, cause no increase in the activity of antioxidant enzymes. Filaments of *C*. *herbarum*, however, are capable of absorbing and accumulating higher amounts of iron(III) ions in the entire range of applied concentrations. Oxidative stress occurs as a result of the influence of high concentrations of iron(III) ions on filaments of *C*. *herbarum*. An increase in the activity of antioxidant enzymes, however, in combination with an increase in the concentration of proteins and monosaccharides in cells, suggests the development of defense mechanisms against the toxic effect of iron(III) ions, which was not observed for the yeast *R*. *mucilaginosa*. According to the literature data, filamentous fungi are distinguished by a high capacity for bioaccumulation of other heavy metals, such as Zn or Cu, which is related to an increase in the enzymatic (superoxide dismutase, catalase, glutathione reductase) and nonenzymatic activities (thiol compounds) of antioxidant systems^7,38,46–48^.

Considering all of the above, iron(III) ions at a concentration of 1 mg/L stimulated cellular metabolism in *R*. *mucilaginosa*. At concentrations of more than 5 mg/L in the environment, the fungus developed a mechanism that inhibited the intake of iron(III) ions. A decrease in the content of proteins and monosaccharides was then observed in yeast cells, as well as an increase in the activity of antioxidant enzymes and an increase in the secretion of proteins and monosaccharides into the medium. Simultaneously, even in the case of high initial concentrations of iron(III) ions in the medium, no decrease in the biomass of yeast was observed. It seems therefore that *R*. *mucilaginosa* can function in waters strongly contaminated with iron(III) ions. A considerably higher possible biosorption of iron(III) ions was observed for the filamentous fungus *C*. *herbarum*. In fungal cells under the influence of high concentrations of iron(III) ions, the appearance of defense mechanisms was observed, which was manifested in a substantial increase in the content of proteins and monosaccharides and an increase in the activity of antioxidant enzymes. Iron(III) ions at a concentration from 25 to 100 mg/L, however, caused a substantial reduction in the fungal biomass. The above results suggest that the fungus can actively accumulate iron(III) ions and therefore remove them from the aquatic environment if the concentration of the ions does not exceed 25 mg/L. It seems to be of high importance from the point of view of the functioning of aquatic ecosystems.

## Materials and Methods

### Species of fungi and culture conditions

The experiment was conducted for two fungi species: the yeast *R*. *mucilaginosa* and *C*. *herbarum*. They are species that most commonly occur in the limnic waters of NE Poland from where they were isolated. The isolated fungi were identified to the species level by means of the Sanger sequencing method, with the application of two primers ITS1 (5′-CTTGGTCATTTAGAGGAAGTAA-3′) and ITS4 (5′TCCTCCGCTTATTGATATGC-3′). ITS4 is a standard fungal primer that is commonly used^49^, but ITS1 was designed in the Fast PCR program. The sequenced products were analyzed with BLAST through the National Center for Biotechnology Information (NCBI) website by aligning input sequences against published nucleotide sequences. The degree of overlap of our fungal strains with fungal strains deposited in GenBank amounted to 99.9% for *R*. *mucilaginosa* (AB916512.1) and 100% for *C*. *herbarum* (LN808882.1). The identification of *C*. *herbarum* was further confirmed by amplification of the actin gene^50^ with primers ACT512f (ATGTGCAAGGCCGGTTTCG) and ACT783r (TACGAGTCCTTCTGGCCCAT)^51^ as a molecular marker to correctly identify this fungus.

Pure colonies of the fungi identified to the species level were recultured in malt extract agar medium (1% malt extract, 1.5% agar). For the purpose of establishing experimental cultures, the fungi were transferred to sterile disposable culture bottles containing 200 mL of liquid medium previously sterilized by autoclaving (1% malt extract, pH 5.0) and enriched with iron(III) ions or without the addition of iron(III) ions (control culture). The acidic pH of the medium prevents the precipitation of introduced iron ions^52^. Iron(III) chloride (anhydrous, powder, ≥99.99% trace metals basis, Sigma-Aldrich) was used for experimental analyzes. In the experimental analysies Each media contained 5 g/L fungi. The iron(III) ion solution was added to culture media to final concentrations from 0.25 to 100 mg/L. The experimental fungal cultures were kept in the dark under stable temperature conditions (25 °C) for 5 days for *R*. *mucilaginosa* and 7 days for *C*. *herbarum*. After that time, cultures of both species were in the same phase of growth, and their biomass growth per time unit was constant. For all chemical analyzes the fungal suspension was filtered through 0.22 µm nitrocellulose filters (Millipore). Throughout the experiment, the fungal cultures were shaken at 160 rpm. After the aforementioned time, cultures of both fungal species were filtered through 0.22 µm nitrocellulose filters (Millipore).

### Determination of fungal biomass

The determination of fresh fungal mass involved weighing the nitrocellulose filters 0.22 µm (Millipore) on an analytical balance and then filtering the suspension through the filters. After the procedure, the filters were dried at room temperature and weighed again.

### Determination of iron(III) concentration in fungal biomass and culture medium

Then, the filters were rinsed three times with distilled water and homogenized in liquid nitrogen. After homogenization, concentrated nitric(V) acid was added. The resulting sample was subjected to UV mineralization. An analogical procedure was performed for the filtrate (medium), where concentrated nitric(V) acid was added, followed by UV mineralization. The iron concentration, both in fungal cells and in the medium, was determined by means of flame atomic absorption spectrometry (FAAS) (AA-7000 Shimadzu).

### Determination of protein concentration in fungal biomass and culture medium

The filters were rinsed three times with distilled water and then homogenized in liquid nitrogen with the addition of 0.1 mol/L sodium hydroxide to isolate proteins from the fungal cells. The concentration of proteins in the resulting extract and culture medium was determined by means of the spectrophotometric method (spectrophotometer, Shimadzu UV-Vis 1201) with the application of Folin’s reagent^53^. The protein concentration was also determined in pure 1% malt extract. The concentration of proteins obtained for both fungal species grown in the culture medium was then adjusted by the resulting value.

### Determination of the concentration of monosaccharides in fungal biomass and culture medium

The filters were rinsed three times with distilled water and then homogenized in liquid nitrogen with the addition of 96% ethanol to isolate monosaccharides from the fungal cells. The concentration of monosaccharides in the resulting extract and culture medium was determined by means of the spectrophotometric method (spectrophotometer, Shimadzu UV-Vis 1201) with the application of arsenomolybdenum reagent^54^. The concentration of monosaccharides was also determined in pure 1% malt extract. The concentration of monosaccharides obtained for culture medium for both fungal species was then adjusted by the resulting value.

### Determination of the activity of catalase and NADH-dependent peroxidase

The filters were rinsed three times with distilled water and then homogenized in liquid nitrogen with the addition of 0.1 mol/L of phosphate buffer (pH 6.0) containing 0.1 mmol/L EDTA and 1% PVP. The homogenate was centrifuged at 2000 rpm for 20 min at 4 °C. The activities of catalase (EC 1.11.1.6) and NADH-dependent peroxidase (EC 1.11.1.1) were determined in the resulting supernatant. The activity of catalase was determined by means of the method of Aebi^55^, which involves monitoring H_2_O_2_ reduction and is expressed as the decrease in absorbance at a wavelength of 240 nm over time (spectrophotometer, Shimadzu UV-Vis 1201). The activity of NADH-dependent peroxidase was determined by the method of Ishida *et al*.^56^, which involves monitoring NADH reduction and is expressed as the decrease in absorbance at a wavelength of 340 nm over time (spectrophotometer, Shimadzu UV-Vis 1201).

### Determination of the activity of superoxide dismutase

The filters were rinsed three times with distilled water and then homogenized in liquid nitrogen with the addition of lysis buffer containing 0.1 mol/L phosphate buffer (pH 7.8), 3 mmol/L magnesium sulfate(VI), 1 mmol/L dithiothreitol (DTT), and 3 mmol/L EDTA. The homogenate was centrifuged at 2000 rpm for 10 min at a temperature of 4 °C. The activity of superoxide dismutase (EC 1.15.1.1) was determined in the obtained supernatant. The activity of superoxide dismutase was determined by means of the method of Beauchamp and Fridovich^57^, which involves determining the degree of reduction of nitroblue tetrazolium (NBT) by superoxide anion that results from photochemical reduction of riboflavin by light measured with the spectrophotometric method at a wavelength of 560 nm (spectrophotometer Shimadzu UV-Vis 1201). The applied method is based on the fact that the reaction of NBT reduction is hindered by superoxide dismutase. One unit of activity of superoxide dismutase was defined as the concentration of the enzyme that hinders the reduction of NBT by 50%.

### Statistical analyses

The experiment was repeated four times at different time periods. Each time, all of the cultures were performed in 3 repetitions. For the purpose of estimating the significance of differences between variables, a Kruskal-Wallis test was applied. All the results are presented as the mean values ± SD. The standard deviation from the mean value of all of the analyzed parameters was not higher than 5%. All statistical analyses were performed with Statistica 7 software.
